# Parents‘ and healthcare professionals’ perception toward the introduction of a new fully liquid hexavalent vaccine in the Malaysian national immunization program: a cross-sectional study instrument development and its application

**DOI:** 10.3389/fimmu.2023.1052450

**Published:** 2023-04-27

**Authors:** Lama Al Bashir, Aniza Ismail, Syed Mohamed Aljunid

**Affiliations:** ^1^ Department of Community Health, Faculty of Medicine, University Kebangsaan Malaysia (UKM), Kuala Lumpur, Malaysia; ^2^ International Centre for Casemix and Clinical Coding, Faculty of Medicine, UKM Medical Centre, National University of Malaysia, Kuala Lumpur, Malaysia; ^3^ Malaysian Health Economic Association (MAHEA), International Centre for Casemix and Clinical Coding, UKM Medical Centre, National University of Malaysia, Kuala Lumpur, Malaysia; ^4^ Department of Health Policy and Management, College of Public Health, Health Science Center, Kuwait University, Kuwait City, Kuwait; ^5^ Department of Community Medicine, School of Medicine, International Medical University, Kuala Lumpur, Malaysia

**Keywords:** fully liquid vaccine, combined vaccine, perception, parents, health care professional, instrument development, national immunization program, Malaysia

## Abstract

A newly developed fully liquid hexavalent vaccine that comprises six antigens for Diphtheria, Tetanus, acellular Pertussis, Inactivated Poliomyelitis, Haemophilus Influenza type b., and Hepatitis B, is proposed to be introduced in the Malaysian national immunization program, instead of the non-fully liquid pentavalent vaccine and monovalent Hepatitis B vaccine that is currently employed in the immunization schedule. Although the introduction of new vaccines is a necessary intervention, it still needs to be accepted by parents and healthcare professionals. Hence, this study aimed to develop three structured questionnaires and to investigate the participants’ perception and acceptability toward the incorporation of the new fully liquid hexavalent vaccine. A cross-sectional study was conducted among a sample of 346 parents, 100 nurses, and 50 physicians attending twenty-two primary health care centers in the states of Selangor and the Federal Territory of Kuala Lumpur and Putrajaya during 2019-2020. The study found that Cronbach’s alpha coefficients for the study instruments ranged from 0.825 to 0.918. Principal components analysis produced a good fit with KMO>0.6. For the parents’ perception questionnaire, the only extracted factor explained 73.9 % of the total variance; for the nurses’ perception toward a non-fully and fully liquid combined vaccine, there was a sole extracted factor that explained 65.2 % and 79.2% of the total variance, respectively. Whereas for the physicians’ perception, there was one factor extracted that explains 71.8 % of the total variance. The median score for all the questionnaire items ranged from 4 to 5 (Q1 and Q3 vary between 3-5). Parents' ethnicity was significantly associated (P-value ≤ 0.05) with the perception that the new hexavalent vaccine would reduce their transportation expenses. Moreover, a significant association (P-value ≤ 0.05) was found between physicians' age and the perception of the hexavalent vaccine's ability to decrease patient overcrowding in primary healthcare centers. The instruments used in this study were valid and reliable. Parents of Malay ethnicity were the most concerned about transportation expenses since they have the lowest income and are more concentrated in rural areas compared to other races. Younger physicians were concerned about reducing patient crowding and hence reducing their workload and burnout.

## Introduction

In healthcare, prevention of disease is always better than cure, which is why immunization and childhood vaccination programs are crucial public health measures that have decreased worldwide morbidity and mortality linked to several pediatric infectious illnesses (1).

Growth in the number of effective vaccines over the last two decades has posed substantial economic and logistical difficulties (2). Thus, current initiatives strive to formulate vaccines that confer protection against various microbes combined in a single multivalent injection (3).

In Malaysia, the current immunization schedule includes several combination vaccines, whereas the non-fully liquid combined vaccine is the broadest. It is a pentavalent vaccine (DTaP–IPV//Hib) that provides immunity to five infectious illnesses: diphtheria, tetanus, acellular pertussis, inactivated poliomyelitis, and *Haemophilus influenzae* type b. This vaccine is administered as three primary doses for children at the age of 2, 3, and 5 months and as a booster dose at the age of 18 months. It is packaged as a pre-filled liquid syringe that contains DTaP-IPV and a vial that contains Hib as a white lyophilized powder that ought to be reconstituted instantly before administration.

Recently, a fully liquid hexavalent combined vaccine (DTaP–IPV–Hib-HepB) was developed. It combines the five antigens for the latter pentavalent vaccine with the hepatitis B antigen together in one injection. It follows the same administration time frame of the pentavalent vaccine for both the primary and booster doses. The fully liquid hexavalent vaccine was authorized by the World Health Organization (WHO) in 2016 (4) and the European Medicines Agency (EMA) in 2015 (5) based on the findings of multiple clinical trials conducted in several countries such as the Republic of Korea in 2017 by Kim et al. (6); Finland, Sweden, and Italy in 2016 (7); Turkey in 2016 (8); Argentina, Mexico, Peru, Costa Rica, and Colombia in 2013 (9); and South Africa in 2011 (10). These clinical trials showed that this fully liquid hexavalent vaccine conferred a high rate of seroprotection/seroconversion after 1 month of the three primer doses and the booster dose and that it had a similar safety profile and generated antibody responses that were comparable to those following separately administered pentavalent and Hep B vaccines. The merits of using a fully liquid vaccine (does not require reconstitution) are that it reduces the vaccine administration time and handling errors (11, 12) and decreases needle stick injury among healthcare professionals (HCPs) who are in charge of administering vaccination (13, 14). In addition, there are benefits of using hexavalent vaccine instead of pentavalent vaccine: it could lessen the number of injections administered to the children and consequently reduce the child and their parents’ stress level (15), it could decrease the adverse events incidence by reducing the number of injections (16), and it could increase vaccine compliance and timeliness (17) and consequently reduce regional disparities in vaccination coverage and ultimately increase global immunization coverage (18).

Given all the mentioned facts that favor the incorporation of the new fully liquid hexavalent combined vaccine into the immunization program and as “the vaccine acceptability” is one of the distinctive factors that form the decision to employ a new vaccine in national vaccination program (19), this study was designed to measure the acceptability and perception of the main persons involved in the immunization process, namely, parents, nurses, and physicians.

Parents of young children are the primary health decision-makers for their children, and their knowledge and acceptance regarding immunization in general and their perception regarding the implementation of new vaccines such as the fully liquid hexavalent vaccine in particular have a great impact on the immunization status of their kids.

Nevertheless, concerning immunization, HCPs’ views and acceptance are considered more significant than the parents’ opinions (20) since their input is vital to the development and execution of the vaccination program. In fact, knowing whether they support the introduction of a new vaccine is crucial (21). Therefore, prior to conducting any alteration of the immunization schedule, it is important to take into account their perspective regarding that (22).

In most countries and mainly in Malaysia, the primary healthcare practitioner in charge of providing vaccinations are registered nurses, and they frequently exercise leadership in creating and maintaining effective immunization programs (23, 24). Nurses’ positive perception toward a new vaccine is associated with their intention to recommend that vaccine. Gilca et al. demonstrated that perceived nurses’ professional support (nurses’ acceptance of a new vaccine) is one of the imperative factors that shape the nurses’ decision to recommend a newly employed vaccine (25).

Regarding physicians, Dube et al. indicated that their knowledge and perception toward a new vaccine is known to impact their willingness to recommend its implementation in the immunization schedule and that their recommendation is a major determinant of vaccine integration in the vaccination program (22). Many previous studies had verified the substantial association between physicians’ perception and acceptance of a certain vaccine and its subsequent uptake and employment in the publicly funded immunization program; these studies were conducted in several countries around the world such as France, United Kingdom and Italy (26, 27), Germany (26–28), Belgium (29), and the United States of America (30, 31). Furthermore, physicians have a major influence on parents’ acceptance of new vaccines. This fact was shown by a representative survey among parents in Germany, where most of them recognized physicians as their main source of information about vaccination (32). Likewise, a survey conducted in Australia indicated that parents’ willingness to have their children receive a vaccine for invasive meningococcal disease was mostly enticed by the recommendation of family physicians (33).

Upon reviewing the literature, there were several questionnaires developed to measure parents’ and/or HCPs’ perceptions or acceptance toward adding a new vaccine in the immunization program, but none of them addressed perceptions toward the fully liquid hexavalent vaccine in particular. Hence, this study aimed to assess parents’ and HCPs’ perceptions regarding the introduction of a fully liquid hexavalent vaccine in the Malaysian national immunization program using newly developed instruments.

## Materials and methods

### Study setting and population

A cross-sectional, descriptive instrument development study was designed. Participants were recruited from 21 primary healthcare centers (PHCs) in the states of Selangor and the Federal Territory of Kuala Lumpur (Malaysia) from February to July 2020. PHCs were chosen by the stratified random sampling method where a list of all centers that provide childhood vaccination services in the earlier mentioned states in Malaysia was prepared and organized as centers for each district, and the number of centers recruited from each district was determined and chosen randomly according to district population percentage for each state.

### Population

A sample size of 350 parents was determined using the Cochrane formula based on percentages drawn from a study conducted recently in Malaysia (34). Parents were selected by systematic sampling where every other parent setting in the Maternal and Child Health Clinic (MCH) waiting area was invited to participate in the study. Only parents who were older than 18 years (old enough to consent to their participation in the study) and whose age of their vaccinated child is between 1 and 24 months were included in the study.

For HCPs, a sample size of 100 nurses and 50 physicians was determined using Pocock’s sample size formula for two proportions, based on percentages drawn from a previous perception study done by Lloyd et al. in 2015 (35). Only HCPs who administer vaccine injection (nurses) or supervise and counsel for the vaccination process (physicians) and who had a practice of 1 year or more in the MCH were recruited. Two sampling methods were used to select them based on the number of HCPs working in that center: first, universal sampling for PHCs that have five or fewer nurses and two or fewer physicians in the MCH; second, systematic sampling for PHCs that have more than five nurses and more than two physicians in the MCH, where a list of HCPs is prepared and only nurses or physicians with odd numbers starting from number 1 in the list was included in the study until reaching the target sample size for that MCH.

Both parents/caregivers and HCPs had to read the study information sheet (**Supplementary Material: Documents 1**, **2**) and sign the informed consent (**Supplementary Material: Document 3**) before filling up their questionnaires. A trained research assistant was assigned to interview parents/caregivers to complete their questionnaires, while the HCPs questionnaire was self-administered.

### Ethical approvals

During all the study stages, the researchers were committed to all ethical considerations required to conduct this research. They attained all the required ethical approvals from (1) the ethical committee of the Faculty of Medicine-National University of Malaysia (UKM) on 29 June 2019 (Ref. No. UKM FPR. 4/244/FF-2019-318) (2), the ethical committee of UKM Center for Research and Instrumentation Management on 28 July 2019 (Ref. No. UKM PPI.800-1/1/5/JEP-2019-406), and (3) the National Medical Research and Ethics Committee (MREC) on 21 August 2019 [Ref. No. KKM/NIHSEC/P19-1482 (6)]. Official approvals were obtained from Selangor and the Federal Territory of Kuala Lumpur State Health Offices in addition to verbal and written approvals from each district’s health office and the doctor in charge of each health center involved in the study. Participation in this research was voluntary and confidential and informed consent was provided and signed by each of the study participants.

### Development of parents’ and healthcare professionals’ perception questionnaire

Instruments assessing parents’ and HCPs’ (nurses and physicians) perceptions were developed through three stages: (1) instruments construction, (2) checking for instruments’ clarity and useability, and (3) evaluating instruments’ validity and reliability.

### Stage 1: Instruments construction

The main components and domain areas for each instrument were determined after reviewing several previous studies to create the preliminary version of the parents’ questionnaire (36–38) and the HCPs’ questionnaire (22, 25, 36). Items listed in the instruments’ initial draft were then discussed with a panel of specialists, which involved public health specialists, pediatricians, family medicine specialists, and biostatisticians. Instruments were written in Bahasa Melayu language, which is the official language of Malaysia. The adjusted version of the instruments focused on the following domains:

#### Parents’ instrument

- Socio-demographic data
- Perception regarding the introduction of the new fully liquid hexavalent vaccine in the national immunization program

#### HCPs’ (nurses) instrument

- Socio-demographic and professional characteristics
- Perception regarding the non-fully liquid pentavalent vaccine administration
- Perception regarding the introduction of the new fully liquid hexavalent vaccine in the national immunization program

#### HCPs’ (physicians) instrument

- Socio-demographic and professional characteristics
- Perception regarding the introduction of the new fully liquid hexavalent vaccine in the national immunization program

### Stage 2: Checking for instruments’ clarity and useability

A pre-test survey was used to check the clarity, usability, and comprehension of the constructed instruments in a sample of parents and HCPs who met the inclusion criteria at one PHC in Selangor/Malaysia. A final version of each instrument was developed containing open and closed items and a five-point Likert scale was used to assess parents’ perception (strongly agree, agree, neutral, disagree, and strongly disagree) (**Supplementary Material: Documents 4**–**6**).

### Stage 3: Evaluating instruments’ validity and reliability

To evaluate the validity and reliability of the instrument’s final version, a pilot study using the final version of each study instrument was conducted.

For construct validity, exploratory factor analysis (EFA) was selected to investigate the internal construct of each questionnaire’s perception items. For factor extraction, principal components analysis (PCA), Varimax rotation, and Kaiser normalization were used (39). Scree test and Kaiser’s criterion based on eigenvalues for the correlation matrix were used for extracting the number of factors for each instrument/scale (40). Conditions for the adequacy of the factor analysis were checked first based on the following (1): a sample size of a minimum of 1:5 items-to-subject ratios for the reliability of the factors emerging as suggested by Chua (41), (2) significant Bartlett’s test of sphericity at α < 0.05, (3) large values of the Kaiser–Meyer–Olkin (KMO) greater than 0.6, (4) the anti-image correlation for all items must be above 0.5, and (5) all items must have a communality that is above 0.4.

The instrument’s reliability was assessed using Cronbach’s alpha coefficient. According to Kline, Cronbach’s alpha value that is more than 0.70 is indicative of adequate internal consistency (42).

### Statistical analysis

Statistical analysis was performed using SPSS 28.0 software. For instrument development, Cronbach’s alpha coefficients to test for internal consistency (reliability) and EFA to test for construct validity were used. Categorical data were expressed as frequency (*n*) and percentage (%) to convey the study’s findings. Data that follow a normal distribution were presented by using mean and standard deviation (SD). For data that were non-normally distributed, the median and the first (Q1) and third (Q3) quartiles were employed. To explore the association between participants’ perceptions and other factors, the Pearson chi-square test of independence was used, with a statistical significance of ≤0.05.

## Results

### Development of parents’ and healthcare professionals’ perception questionnaire

#### Reliability

Cronbach’s alpha coefficients ranged from 0.825 to 0.918 for each scale of the instruments (**Supplementary Material: Document 7; Table 1**).

#### Construct validity

For the study sample size, the ratio of a sample size to the number of items for parents’ fully liquid hexavalent vaccine perception scale was 1:69, while for nurses’ non-fully liquid pentavalent and fully liquid hexavalent vaccine perception scales, the ratio was 1:25 and 1:33, respectively. Physicians’ fully liquid hexavalent vaccine perception scale had a ratio of 1:8.

The study’s four scales had KMO test values ranging from 0.678 to 0.881, indicating that factor analysis could be performed, and all the significance values of Bartlett’s test of sphericity were less than 0.001, indicating the suitability of the data for factor analysis. Furthermore, the communalities of the items in the parents’ fully liquid hexavalent vaccine perception scale ranged from 0.623 to 0.826, and the anti-image correlation (0.838–0.922). For the nurses’ non-fully liquid pentavalent and fully liquid hexavalent vaccine perception scales, the communalities of their items ranged from 0.490 to 0.830 and from 0.686 to 0.848, respectively, and their anti-image correlation range was 0.634–0.769 and 0.660–0.754, respectively. Finally, physicians’ fully liquid hexavalent vaccine perception scale items had a communalities range of 0.616–0.842 and an anti-image correlation range of 0.689–0.943 (**Supplementary Material: Document 7; Table 2**).

Given these overall indicators, EFA was then conducted for the four scales using PCA extraction and Varimax rotation based on an eigenvalue of greater than 1 and a minimum factor loading cutoff point of 0.4. The PCA extracted only one component in each scale and that is why the Varimax rotation matrix cannot be computed. Component 1 in parents’ fully liquid hexavalent vaccine perception scale had a total variance of 3.697 (initial eigenvalue greater than 1) and explains 73.9% of the variance, while component 1 in nurses’ non-fully liquid pentavalent and fully liquid hexavalent vaccine perception scales had a total variance of 2.6 and 2.3 and explain 65.2% and 79.2% of their variance, respectively. For the physicians’ fully liquid hexavalent vaccine perception scale, component 1 had a total variance of 4.3 and explains 71.8% of its variance (**Supplementary Material: Document 7; Table 3**).

The minimum factor loading cutoff point for this study was 0.4. Based on the EFA, all the items in the study scales had a factor loading of ≥0.4. The factor analysis results have shown that five items were loaded onto factor 1 (0.789–0.901), which measures “parents’ perception toward fully liquid hexavalent vaccine”, four items were loaded onto factor 1 (0.700–0.911), which measures “nurses’ perception toward non-fully liquid pentavalent vaccine”, and three items were loaded onto factor 1 (0.828–0.921), which measures “nurses’ perception toward fully liquid hexavalent vaccine”. For “physician’s perception toward fully liquid hexavalent vaccine”, six items were loaded onto factor 1 (0.785–0.918) (**Supplementary Material: Document 7; Table 4**).

### Participants’ socio-demographic and professional characteristics

A total of 346 parents (response rate, 98.8%) participated in the perception study, where the minimum age was 19 years, and the maximum age was 59 with a mean age of 31.97. Most of the parents were women (70.5%), and most of them were the mothers of the vaccinated child (68.8%), while 1.7% were caregivers. More than half of the parents have post-secondary education and 35% of them are working in the private sector and 26% were housewives (**Supplementary Material: Document 7; Table 5**).

For nurses, 100 participated in the study; their mean age and experience in childhood vaccination were 34 and 8 years, respectively. Community health nurses constitute 61% of the nurses who participated in the study while 39% were staff nurses. Ninety-seven percent stated that their role was to prepare and administer injections only (**Supplementary Material: Document 7; Table 6**).

For physicians, 50 participated in the study, where their mean age was 33 years, and had an average experience of 4.5 years in the childhood vaccination field. Most of the physicians were female (88%) and all of them were medical officers; 42.8% stated that their role was to counsel parents regarding childhood vaccines and to supervise the process of vaccination (**Supplementary Material: Document 7; Table 7**).

### Participants’ perception and its associated factors

Study participants’ median score for each perception scale and its items, and first quartile (Q1) and third quartile (Q3) scores are depicted in **Table 1**. Parents’ fully liquid hexavalent vaccine perception scale and all its items had a total median score of 4 (agree), and the scale and its items’ first quartile score were also 4, while its Q3 was 5 (strongly agree). For the nurses’ non-fully liquid pentavalent vaccine perception scale, it had a total median score of 4, while its Q1 and Q3 scores were 3 and 4.5, respectively. All these scale items had a median score of 4. The median score for nurses’ fully liquid hexavalent vaccine perception scale and two of its items (in terms of supporting Hexaxim employment and that it will reduce the staff workload) was 5. For the physicians’ fully liquid hexavalent vaccine perception scale, the total median score was 4 and most of the item median, Q1, and Q3 were 4, 4, and 5, respectively.

**Table 1:** Median score of participants’ perception for each scale and its items.

Instrument scale/Item	Participants’ perception score
	Median (Q1–Q3)*
Parents’ fully liquid hexavalent vaccine perception scale [Total median score: 4, Q1: 4, Q3: 5]	
The fully liquid hexavalent vaccine could reduce a child**’**s pain and discomfort	4 (4–5)
The fully liquid hexavalent vaccine could reduce the number of visits	4 (4–5)
The fully liquid hexavalent vaccine could reduce transportation expenses	4 (4–5)
The fully liquid hexavalent vaccine could increase compliance	4 (4–5)
The current immunization schedule should be reviewed	4 (4–5)
Nurses’ non-fully liquid pentavalent vaccine perception scale [Total median score: 4, Q1: 3, Q3: 4.5]	
Non-fully liquid pentavalent vaccine reconstitution could be a time loss	4 (3–4)
Non-fully liquid pentavalent vaccine reconstitution has too many steps	4 (4–5)
Non-fully liquid pentavalent vaccine reconstitution could lead to handling error	4 (4–5)
Non-fully liquid pentavalent vaccine reconstitution could lead to needle stick injury	4 (3–5)
Nurses’ fully liquid hexavalent vaccine perception scale [Total median score: 5, Q1: 4, Q3: 5]	
The fully liquid hexavalent vaccine could reduce staff workload	5 (4–5)
The fully liquid hexavalent vaccine could reduce patient overcrowding	4.5 (4–5)
Support fully liquid hexavalent vaccine introduction to NIP	5 (4–5)
Physicians’ fully liquid hexavalent vaccine perception scale [Total median score: 4, Q1: 4, Q3: 5]	
Parents could be interested in replacing fully liquid hexavalent with pentavalent vaccine	4 (3–5)
The fully liquid hexavalent vaccine could lead to cost savings	4 (4–5)
The fully liquid hexavalent vaccine could reduce patient overcrowding	4.5 (4–5)
The fully liquid hexavalent vaccine could ease the incorporation of other vaccines such as PCV	4 (4–5)
The fully liquid hexavalent vaccine could enhance compliance with the immunization schedule	4 (4–5)
Support fully liquid hexavalent vaccine introduction to NIP	5 (4–5)

*1 = strongly disagree, 2 = disagree, 3 = neutral, 4 = agree, 5 = strongly agree.

In **Tables 2**–**4**, the association between participants’ perception and their socio-demographic and professional characteristics was explored using the Pearson chi-square test of independence (*p*-value ≤ 0.05). The study found that there was no significant association between most of the four scale items and the socio and clinical practice profile factors for the study participants. Parents’ ethnicity in **Table 2** was associated with the perception that a fully liquid hexavalent vaccine would reduce their transportation expenses to and from the vaccination center (*p*-value = 0.035). Moreover, **Table 4** shows that there was a significant association between age and physicians’ perception regarding fully liquid hexavalent vaccine that it could lessen patient overcrowding in the MCH (*p*-value = 0.035).

**Table 2:** Factors associated with parents’ fully liquid hexavalent vaccine perception (*n* = 346).

Perception Scale/Item	*p*-value*				
	Age	Gender	Ethnicity	Education	Occupation
The fully liquid hexavalent vaccine could reduce my child**’**s pain and discomfort	0.09	0.383	0.992	0.569	0.797
The fully liquid hexavalent vaccine could reduce the number of visits	0.834	0.261	0.908	0.906	0.906
The fully liquid hexavalent vaccine could reduce transportation expenses	0.559	0.170	0.035**	0.487	0.487
The fully liquid hexavalent vaccine could increase compliance	0.555	0.243	0.562	0.559	0.559
The current immunization schedule should be reviewed	0.730	0.634	0.218	0.825	0.825

*Tested using Pearson chi-square test of independence. **Significant finding (p-value ≤ 0.05).

**Table 3:** Factors associated with nurses’ non-fully liquid pentavalent and fully liquid hexavalent vaccine perception (*n* = 100).

Perception Scale/Item	*p*-Value*			
Nurses’ non-fully liquid pentavalent vaccine perception scale	Age	Profession	Role	Experience
Non-fully liquid pentavalent vaccine reconstitution could be a time loss	0.278	0.588	0.063	0.157
Non-fully liquid pentavalent vaccine reconstitution has too many steps	0.528	0.163	0.484	0.336
Non-fully liquid pentavalent vaccine reconstitution could lead to handling error	0.856	0.654	0.123	0.574
Non-fully liquid pentavalent vaccine reconstitution could lead to needle stick injury	0.956	0.531	0.333	0.077
Nurses’ fully liquid hexavalent vaccine perception scale				
The fully liquid hexavalent vaccine could reduce staff workload	0.313	0.925	0.989	0.893
The fully liquid hexavalent vaccine could reduce patient overcrowding	0.545	0.492	0.977	0.402
Support fully liquid hexavalent vaccine introduction to NIP	0.161	0.572	0.997	0.502

*Tested using Pearson chi-square test of independence.

**Table 4:** Factors associated with physicians’ fully liquid hexavalent vaccine perception (*n* = 50).

Perception Scale/Item	*p*-value*			
	Age	Gender	Role	Experience
Parents could be interested in replacing fully liquid hexavalent with pentavalent vaccine	0.059	0.741	0.474	0.566
The fully liquid hexavalent vaccine could lead to cost savings	0.307	0.828	0.206	0.867
The fully liquid hexavalent vaccine could reduce patient overcrowding	0.035**	0.473	0.684	0.449
The fully liquid hexavalent vaccine could ease the incorporation of other vaccines such as PCV	0.287	0.297	0.833	0.539
The fully liquid hexavalent vaccine could enhance compliance with the immunization schedule	0.106	0.553	0.559	0.581
Support fully liquid hexavalent vaccine introduction to NIP	0.185	0.372	0.413	0.829

*Tested using Pearson chi-square test of independence. **Significant finding (p-value ≤ 0.05).

## Discussion

As new vaccines are being developed and expected to be introduced soon in the vaccination program, it is essential to inspect the acceptance and perception toward the implementation of new vaccinations among parents of young children, as well as among the HCPs who will recommend and/or administer these vaccines (36). Moreover, according to Bakhache et al. (36), “Governmental bodies and healthcare policymakers who formulate recommendations as part of national immunization programs are reciprocally influenced by the guidance from the medical community, typically *via* specific expert advisory panels as well as by the likelihood that the general public will accept and support a potential new vaccine” (36).

To date, there are no standardized instruments to assess both parents’ and HCPs’ perceptions and acceptability toward fully liquid hexavalent combined vaccine introduction in the national immunization program in Malaysia and other countries across the world. Therefore, this study addressed the development and validation of such instruments and demonstrated their perception findings.

### Development of parents’ and healthcare professionals’ perception questionnaire

Our study used a panel of specialists to review each scale content in terms of its conformity to the study objectives and domains and to check for clarity and comprehension of items and the results of the EFA proved that the specialists’ team attained the core content of each scale. The newly developed perception instruments had an excellent psychometric property, where, according to their internal consistency and construct validity results, the study instruments were both reliable and valid.

A recent similar study was conducted in Malaysia (2017) to validate the Malay version of the parent’s attitude toward the childhood vaccination questionnaire (43). The sample population was 151 Malaysian parents attending the MCH unit of a PHC located in Klang Valley. The questionnaire Kaiser–Meyer–Olkin (KMO) value was 0.736, which is comparable to the KMO values of our study scales. Moreover, the overall questionnaire Cronbach’s alpha was 0.77, which was lower than the Cronbach’s alpha generated in all of our study scales.

Another validation study was carried out in Ghana (2019) to investigate parents’ perception and behavior toward childhood vaccination (44) where they performed a cluster survey of 373 households with children 12–35 months old, using a 22-item survey. They used EFA to accomplish construct validity and Cronbach’s alpha coefficients to check for reliability, where KMO was 0.58 and Cronbach’s alpha for the latter study five scales ranged from 0.41 to 0.87, which was lower than our study values.

### Participants’ perception and its associated factors

In Malaysia, the decision of incorporating a new vaccine into the national immunization program is issued by the Ministry of Health. Even though the decision-making process for implementing a new perspective vaccination program can be expedited by various frameworks and assessment criteria (45), community members such as parents’ and HCPs’ knowledge, perception, and acceptance regarding new vaccination program are known to have an impact on the decision-makers’ intention of adopting a new vaccine (46).

Our findings showed that parents had a positive perception toward the fully liquid hexavalent vaccine’s potential ability to reduce their child’s pain and discomfort, number of visits, and consequently their transportation expenses due to minimizing the number of injections administered to infants/toddlers if Hexaxim was incorporated in the NIP. According to parents’ beliefs, this could lead to increased compliance from their side to the vaccination program, which is compatible with what Kalies et al. found in 2006, where the use of higher valent combination vaccine enhanced parents compliance evinced by timely and complete protection against the infectious diseases covered by the immunization program (17).

Malaysia is a multi-ethnic country in which the Malay ethnic group constitutes the highest proportion of the population followed by Chinese and then Indian and other minority races. This study has shown that the parents’ “Malay ethnicity” was significantly associated with their belief that the fully liquid hexavalent vaccine could reduce their transportation expenses to and from the vaccination clinic due to fewer vaccination visits. This result can be attributed to the fact that Malay has the lowest income and is more concentrated in rural areas compared to richer urban non-Malay (47), which means that they will pay more for transportation expenses.

Our study found that nurses had a negative perception towards the non-fully liquid pentavalent vaccine, which is still currently used in the PHCs across Malaysia. They believed that its reconstitution could mean loss of time and that it has so many steps that could increase the possibility of handling errors and needle stick injury. These findings were similar to a study conducted by Esteve et al. in 2018 to assess the preferences of Spanish nurses for the fully liquid hexavalent vaccine (48), where 74.1% believed that a fully liquid hexavalent vaccine that is ready to use is quicker and simpler compared with non-fully liquid pentavalent vaccines that require reconstitution. Nurses indicated that the time saved by using a fully liquid hexavalent vaccine during the immunization process might be used to carry out additional tasks, such as teaching and enlightening the parents and answering any of their queries about vaccination. Moreover, the majority (87.6%) of nurses were concerned about the use of lyophilized vaccines that need to be reconstituted. Among the concerns for the lyophilized vaccine, 52.2% of nurses expressed their concern about the risk of handling errors during vaccine administration, while 44.8% of nurses were worried about the possibility of needle contamination.

On the contrary, our study showed that nurses had a positive perception towards the fully liquid hexavalent vaccine since it may decrease patient overcrowding in the mother and child healthcare unit and consequently alleviate the nurse’s workload. That is why most nurses supported switching to this new fully liquid vaccine in the Malaysia NIP. This was compatible with a previous study that found that 50% of nurses preferred a fully liquid hexavalent vaccine over one that need to be reconstituted. According to Lloyd et al., “the use of fully liquid vaccines perhaps has more impact on the workload of nurses responsible for the preparation of vaccines” (35). Another survey conducted on 200 MCH nurses who worked for the National Health Services in the UK demonstrated that fully liquid vaccine administration saved almost 5 min over three doses compared to a non-fully liquid vaccine (49). Likewise, De Coster et al. showed that the time required to administer a fully liquid hexavalent vaccination was 50% less than the time required to administer a non-totally liquid hexavalent vaccine in a sample of 96 nurses and pediatricians. These time savings can be quite substantial when considered for the vaccination program on a national level (12).

Concerning physicians’ opinions and perceptions toward the fully liquid hexavalent vaccine, it also gained a high amount of support for its application in the immunization schedule. Physicians had many reasons for the unanimous support for the fully liquid hexavalent vaccine since it could probably lead to cost savings for the governmental bodies that provide the cost of the immunization program as shown in an economic evaluation study conducted by Aljunid et al. (50) where they demonstrated that the fully liquid hexavalent vaccine could reduce the cost per dose and birth cohort if it replaced the non-fully liquid pentavalent and monovalent hepatitis B vaccines in the Malaysian NIP, which would yield significant overall cost savings to the healthcare provider in Malaysia (50).

Other reasons that encouraged physicians in our study to support the fully liquid hexavalent vaccine were that it could ease the incorporation of other vaccines such as PCV, reduce patient overcrowding in the MCH units, and enhance parents’ compliance with the immunization schedule as the parents in our study agreed on, as mentioned earlier.

Furthermore, the study discovered that there was a significant association between physicians’ age and their perception that the fully liquid hexavalent vaccine implementation could reduce patient overcrowding in the MCH unit in the PHCs. This means that younger physicians in our study thought that this new vaccine is a way of reducing patient crowding and hence the daily workload and burnout they have to deal with. This was confirmed by previous studies that reported that the burnout level was higher among young and less experienced physicians (51).

## Limitations

First, data obtained from this study were gathered only from the states of Selangor and the federal territory of Kuala Lumpur PHCs; therefore, the findings may not be generalizable to the rest of Malaysian states. Second, the instruments were developed as a perception measure among parents, nurses, and physicians in Malaysia, and it suits them the most, as these instruments were designed mainly for the Malaysian healthcare system and its national immunization program and wording customs were in Bahasa Melayu language. This could limit the utilization of the instruments in other countries without further translation, refinement, and testing. Third, the study was conducted in the PHCs that belong to the Ministry of Health under the public sector, which might limit the feedback from the HCPs and parents who attended the private healthcare clinics in the two states recruited from Malaysia.

## Conclusion

This study developed and tested three different instruments targeting parents, nurses, and physicians, which measured their perception regarding the employment of a new fully liquid hexavalent combined vaccine in the Malaysian national immunization program. Reliability and validity assessment indicated that the study-developed instruments had a robust psychometric property. The study findings showed that the majority of study participants (parents and HCPs) had a positive perception toward fully liquid hexavalent vaccine introduction in the immunization program.

## Recommendations

The study developed valid and reliable instruments to assess perception regarding the implementation of the fully liquid hexavalent vaccine in the immunization schedule. The three newly developed instruments might assist healthcare policymakers to comprehend the various aspects of parents’ and HCPs’ perceptions regarding the introduction of a new vaccine, which underpins their acceptance and compliance with the amended vaccination program.

## Data availability statement

The raw data supporting the conclusions of this article will be made available by the authors, without undue reservation.

## Ethics statement

The studies involving human participants were reviewed and approved by University Kebangsaan Malaysia ethics committee (ethics committee approval reference # FF-2019-318), Malaysian Ministry of Health Medical Research and Ethics Committee (ethics committee approval reference# NMRR-19-1370-47,862). The patients/participants provided their written informed consent to participate in this study.

## Author contributions

Conceptualization: AI, SA and LB. Formal analysis: AI, SA and LB. Investigation: LB, AI and SA. Methodology: LB, AI and SA. Supervision: AI and SA. Visualization: AI and SA. Writing—original draft: LB. Writing—review and editing: LB, AI and SA. All authors contributed to the article and approved the submitted version.
