# Supplementary material for: Parents‘ and healthcare professionals’ perception toward the introduction of a new fully liquid hexavalent vaccine in the Malaysian national immunization program: a cross-sectional study instrument development and its application
Source: Front Immunol. 2023 Apr 27;14:1052450. doi: 10.3389/fimmu.2023.1052450 (PMC10172506; doi:10.3389/fimmu.2023.1052450)
Supplement: Supplementary file 1 [file Table_1.docx]

**Parents’ And Health Care Professionals’ Perception Toward The Introduction Of A New Fully Liquid Combined Vaccine In The Malaysian National Immunization Program: A Cross-Sectional Study Instruments Development And Its Application**

**Appendix: Supplementary Material**

**Table of contents Page**

Document 1: Parents’ Information Sheet 2

Document 2: HCPs’ Information Sheet 5

Document 3: Participants’ Informed Consent 8

Document 4: Parents’ Questionnaire 9

Document 5: Nurses’ Questionnaire 11

Document 6: Physicians’ Questionnaire 13

Document 7: Study Results Tables 15

- Table 1: Internal consistency (Reliability) of the study instruments 16
- Table 2: Kaiser-Meyer-Olkin, Bartlett’s Test, communalities, and 16

anti-image correlation for the study scale

- Table 3: Summary of total variance explained in Exploratory Factor 17

Analysis (EFA) for study scales

- Table 4: Factor loading based on a principal component analysis for 17

the study scales

- Table 5: Socio-demographic characteristics of parents who participated 17

in the study

- Table 6: Socio-demographic and professional characteristics of nurses 19
- Table 7: Socio-demographic and professional characteristics of physicians 19

**Document (1): Parents’ Information Sheet**

1. **Title of study**: Parents’ and health care professionals’ perception toward the introduction of a new fully liquid combined vaccine in the Malaysian national immunization program: a cross-sectional study instruments development and its application
2. **Name of investigator and institution:**

- Syed Mohamed Aljunid Bin Syed Junid (UKM)
- Aniza Ismail (UKM)
- Lama Saleh Al Bashir (UKM)
- Azimatun Noor Aizuddin (UKM)
- Amrizal Muhammad Nur (UKM)
- Siti Athirah Zafirah binti Abdul Rashid (UKM)

1. **Name of sponsor**: Malaysian Health Economics Association (MAHEA)
2. **Introduction:**

You are invited to participate in a research study because you are the caregiver or parent of a child who is eligible to receive Pentavalent or Hepatitis B vaccines.

The details of the research trial are described in this document. It is important that you understand why the research is being done and what it will involve. Please take your time to read through and consider this information carefully before you decide if you are willing to participate. Ask the study staff if anything is unclear or if you like more information. After you are properly satisfied that you understand this study and that you wish to participate, you must sign this informed consent form.

Your participation in this study is voluntary. You do not have to be in this study if you do not want to. Your refusal to participate will not affect any medical or health benefits to which you are otherwise entitled.

This study has been approved by the Medical Research and Ethics Committee, Ministry of Health Malaysia.

1. **What is the purpose of the study?**

The current immunization schedule implemented at governmental clinics in Malaysia includes a non-fully liquid pentavalent vaccine, which is a combination vaccine that protects against 5 diseases which are diphtheria, tetanus, pertussis, poliomyelitis, and Haemophilus influenza type b. Hepatitis B is not included in the Pentavalent vaccine and is administered as a separate injection.

- The pentavalent vaccine requires 3 primer doses (2-, 3-, and 5 months old infant ) and a booster dose at 18 months old toddler
- HepB requires 3 doses (at birth,1, and 6 months old infants)

Recently, a new and advanced fully liquid vaccine is available and tested worldwide and could replace the pentavalent vaccine in the future; it is a fully liquid hexavalent vaccine that protects against 6 diseases combined in one injection; these diseases are Diphtheria, Tetanus, Pertussis, Poliomyelitis, Haemophilus influenza type b, and Hepatitis B.

- The hexavalent vaccine requires primer doses at (2,3 and 4 or 3,4 and 5 or 2,4- and 6-months old infant) and a booster dose at 24 months old toddler.

The purpose of this study is to investigate Health care professionals' and parents’ perceptions regarding the fully liquid hexavalent combined vaccine introduction in the Malaysian national immunization program.

A total of 350 subjects like you from various Primary Health Care centers across the states of Selangor and the Federal Territory of Kuala Lumpur will be participating in this study.

1. **What kind of study products will I receive?**

A questionnaire

1. **What will happen if I decide to take part?**

You will be interviewed by the study researchers for about 10-20 mins in the vaccination department waiting area, there will be 22 questions and you have to give the answer that feels the most appropriate to you. There will be no expenses paid by you if you decide to participate in this study. The researchers will provide pens and paper to you.

1. **When will I receive the trial product and how should it be kept?**

Not relevant

1. **What are my responsibilities when taking part in this study?**

You must answer all of the questions asked by the study staff honestly and completely.

1. **What kind of treatment will I receive after I participate in the trial?**

Not relevant

1. **What are the potential risks and side effects of being in this study?**

Not Relevant

1. **What are the benefits of being in this study?**

There may or may not be any benefits to you. Information obtained from this study will help the decision maker to replace the currently used Pentavalent and Hepatitis B vaccines with a more economic yet with the same efficient Hexavalent Vaccine. There will be no financial benefit since you will not be paid for your participation in this study.

1. **What if I am injured during this study?**

Not relevant

1. **What are my alternatives if I do not participate in this study?**

You do not have to participate in this study to get your child vaccinated today.

1. **Who is funding the research?**

This study is sponsored by Malaysian Health Economic Association (MAHEA)

1. **Can the research or my participation be terminated early?**

Not Relevant

1. **Will my personal information be kept private?**

All your personal information obtained in this study will be kept and handled with confidentially, following applicable laws and/or regulations. When publishing or presenting the study results, your identity will not be revealed without your expressed consent. Individuals involved in this study and your medical care, qualified monitors and auditors, the sponsor or its affiliates, and governmental or regulatory authorities may inspect and copy your medical records, where appropriate and necessary.

1. **Who should I call if I have questions?**

If you have any questions about the study, please contact the study conductor, Lama Al Bashir, at mobile number: 01128798394

If you have any questions about your rights as a participant in this study, please contact the Secretary, Medical Research & Ethics Committee, Ministry of Health Malaysia, at telephone number 03-2287 4032.

**Document (2): HCPs’ Information Sheet**

1. **Title of study**: Parents’ and health care professionals’ perception toward the introduction of a new fully liquid combined vaccine in the Malaysian national immunization program: a cross-sectional study instruments development and its application
2. **Name of investigator and institution:**

- Syed Mohamed Aljunid Bin Syed Junid (UKM)
- Aniza Ismail (UKM)
- Lama Saleh Al Bashir (UKM)
- Azimatun Noor Aizuddin (UKM)
- Amrizal Muhammad Nur (UKM)

1. **Name of sponsor**: Malaysian Health Economics Association (MAHEA)
2. **Introduction:**

You are invited to participate in a research study because you are dealing daily with Pentavalent and Hepatitis B vaccines prescribing and/or reconstitution and/or administering.

The details of the research trial are described in this document. It is important that you understand why the research is being done and what it will involve. Please take your time to read through and consider this information carefully before you decide if you are willing to participate. Ask the study staff if anything is unclear or if you like more information. After you are properly satisfied that you understand this study and that you wish to participate, you must sign this informed consent form. Your participation in this study is voluntary. You do not have to be in this study if you do not want to. Your refusal to participate will not affect any medical or health benefits to which you are otherwise entitled. This study has been approved by the Medical Research and Ethics Committee, Ministry of Health Malaysia.

1. **What is the purpose of the study?**

The current immunization schedule implemented at governmental clinics in Malaysia includes a non-fully liquid pentavalent vaccine, which is a combination vaccine that protects against 5 diseases which are diphtheria, tetanus, pertussis, poliomyelitis, and Haemophilus influenza type b. Hepatitis B is not included in the Pentavalent vaccine and is administered as a separate injection.

- The pentavalent vaccine requires 3 primer doses (2-, 3-, and 5 months old infant ) and a booster dose at 18 months old toddler
- HepB requires 3 doses (at birth,1, and 6 months old infants)

Recently, a new and advanced fully liquid vaccine is available and tested worldwide and could replace the pentavalent vaccine in the future; it is a fully liquid hexavalent vaccine that protects against 6 diseases combined in one injection; these diseases are Diphtheria, Tetanus, Pertussis, Poliomyelitis, Haemophilus influenza type b, and Hepatitis B.

- The hexavalent vaccine requires primer doses at (2,3 and 4 or 3,4 and 5 or 2,4- and 6-months old infant) and a booster dose at 24 months old toddler.

The purpose of this study is to investigate Health care professionals' and parents’ perceptions regarding the fully liquid hexavalent combined vaccine introduction in the Malaysian national immunization program.

A total of 150 subjects like you from various Primary Health Care centers across the states of Selangor and the Federal Territory of Kuala Lumpur will be participating in this study.

1. **What kind of study products will I receive?**

A questionnaire

1. **What will happen if I decide to take part?**

You will be interviewed by the study researchers for about 20 mins in the vaccination department, there will be 16 questions and you have to give the answer that feels the most appropriate to you. There will be no payment paid to you in this study and you will not be asked to pay anything to the researcher if you decide to participate in this study.

1. **What are my responsibilities when taking part in this study?**

You must answer all of the questions asked by the study staff honestly and completely.

1. **What are the potential risks and side effects of being in this study?**

There are no risks or side effects associated with participating in this study

1. **What are the benefits of being in this study?**

There may or may not be any benefits to you directly. Information obtained from this study may help the decision-makers to design a better vaccination program for Malaysia.

1. **What are my alternatives if I do not participate in this study?**

You do not have to participate in this study if you do not want to, there will be no consequence that will affect you or your job.

1. **Who is funding the research?**

This study is sponsored by Malaysian Health Economic Association (MAHEA)

1. **Will my personal information be kept private?**

All your personal information obtained in this study will be kept and handled in a confidential manner, in accordance with applicable laws and/or regulations. When publishing or presenting the study results, your identity will not be revealed without your expressed consent. Individuals involved in this study and your medical care, qualified monitors and auditors, the sponsor or its affiliates, and governmental or regulatory authorities may inspect and copy your medical records, where appropriate and necessary.

1. **Who should I call if I have questions?**

If you have any questions about the study, please contact the study conductor, Lama Al Bashir, at mobile number: 01128798394

If you have any questions about your rights as a participant in this study, please contact The Secretary, Medical Research & Ethics Committee, Ministry of Health Malaysia, at telephone number 03-2287 4032.

**Document (3): Participants’** **Informed Consent Form**

**Title of Study:** Parents’ and health care professionals’ perception toward the introduction of a new fully liquid combined vaccine in the Malaysian national immunization program: a cross-sectional study instruments development and its application

By signing below, I confirm the following:

- I have been given oral and written information for the above study and have read and understood the information given.
- I have had sufficient time to consider participation in the study and have had the opportunity to ask questions and all my questions have been answered satisfactorily.
- I understand that my participation is voluntary and I can at any time freely withdraw from the study without giving a reason and this will in no way affect my future treatment. I am not taking part in any other research study at this time. I understand the risks and benefits, and I freely give my informed consent to participate under the conditions stated. I understand that I must follow the study doctor’s (investigator’s) instructions related to my participation in the study.
- I will receive a copy of this subject information/informed consent form signed and dated to bring home.
- I will receive a copy of the study result as requested after contacting the study conductor (Lama Al Bashir, email: [P81334@ukm.edu.my](mailto:P81334@ukm.edu.my) )

**Subject:**

| Signature: |  | I/C number: |  |
| --- | --- | --- | --- |
| Name: |  | Date: |  |

**An investigator conducting informed consent:**

| Signature: |  | I/C number: |  |
| --- | --- | --- | --- |
| Name: |  | Date: |  |

**Impartial witness:** *(Required if the subject is illiterate and contents of the participant information sheet are orally communicated to the subject)*

| Signature: |  | I/C number: |  |
| --- | --- | --- | --- |
| Name: |  | Date: |  |

**Document (4): Parents’ Questionnaire**

Section A: Parents' socio-demographic profile

Instructions: please tick the appropriate answer in the box and fill when necessary

**1. Age of the caregiver/parent:** years

**2. Gender**

Male Female

**3. Ethnicity:**

Malay Chinese

Indian Other, please specify: ______________

**4.** **Relationship with the child**

Mother Father Family member Guardian

**5. Marital status**

Single Married Divorced Widowed

**6. Educational level**

Primary or less Secondary Post-secondary

**7. Occupation**

Public sector Private sector Self-employed Unemployed

Housewife

**8. Age of your child to be vaccinated today:** Days/weeks/Months/years

**9. You are attending this clinic today so that your child gets:**

DTaP-IPV/Hib vaccine Hepatitis B vaccine Mono Measles

MMR (Measles, Mumps & Rubella) DTaP-IPV/Hib + Hep B

**10. If you are working, how much are your monthly earnings?** RM

Section B: Parents/Caregivers' Perceptions regarding employment of fully liquid hexavalent combined vaccine in the national immunization program

**11.** **Fully liquid hexavalent combined vaccine could reduce my child’s pain compared with two injections that contain the same number of vaccines.**

Strongly agree Agree Neutral

Disagree Strongly disagree

**12. Fully liquid hexavalent combined vaccine could reduce the number of visits I have to make to the vaccination clinic compared with two injections that contain the same number of vaccines.**

Strongly agree Agree Neutral

Disagree Strongly disagree

**13. Fully liquid hexavalent combined vaccine could reduce my transportation expenses compared with two injections that contain the same number of vaccines.**

Strongly agree Agree Neutral

Disagree Strongly disagree

**14. Fully liquid hexavalent combined vaccine could lead to higher compliance with the vaccination schedule compared with two injections that contain the same number of vaccines.**

Strongly agree Agree Neutral

Disagree Strongly disagree

**15. In case there is an alternative vaccine available, I believe that the current immunization schedule needs to be reviewed:**

Strongly agree Agree Neutral

Disagree Strongly disagree

**Document (6): Nurses’ Questionnaire**

Section A: Nurses' demographic and clinical practice profile

Instructions: Please tick the appropriate answer in the box and fill when necessary

1. **Age**: years

2. **Gender**:

Male Female

3. **Profession**:

Staff Nurse Community Health Nurse

**4.** **Current role in the vaccination process:**

Only prepare injections Only administer injections

Prepare and administer injections Only prescribes vaccines

Prescribe, prepare, and administer a vaccine

**5.** **Years of experience** **in childhood vaccination**:

Section B: Nurses' perception regarding non-fully liquid pentavalent vaccine

**6. I think that** **non-fully liquid pentavalent vaccine reconstitution is a time loss that can be spent with parents/infants:**

Strongly agree Agree Neutral

Disagree Strongly disagree

**7. I think that non-fully liquid pentavalent vaccine reconstitution requires too many steps compared to other vaccines** (such as Hep B)**:**

Strongly agree Agree Neutral

Disagree Strongly disagree

**8.** **I think that** **non-fully liquid pentavalent vaccine reconstitution introduces the risk of handling errors** (such as inadequate shaking of the vial after reconstitution or not aspiring the whole content of the reconstituted vial):

Strongly agree Agree Neutral

Disagree Strongly disagree

**9.** **I think that** **non-fully liquid pentavalent vaccine reconstitution introduces the risk of needle stick injury compared with other vaccines** (such as Hep B)

Strongly agree Agree Neutral

Disagree Strongly disagree

Section C: Nurses' perception toward the introduction of the fully liquid hexavalent combined vaccine in the national immunization program

**10. I think that introducing the fully liquid hexavalent vaccine instead of the non-fully liquid pentavalent and Hepatitis B vaccines in the national immunization program could reduce the daily work burden borne by nurses**

Strongly agree Agree Neutral

Disagree Strongly disagree

**11. I think i****ntroducing the fully liquid hexavalent vaccine instead of the non-fully liquid pentavalent and Hepatitis B vaccines in the national immunization program could decrease the daily patient influx to the vaccination facility**

Strongly agree Agree Neutral

Disagree Strongly disagree

**12 . I would be interested in introducing the fully liquid hexavalent vaccine instead of the non-fully liquid pentavalent and Hepatitis B vaccines in the national immunization program.**

Strongly agree Agree Neutral

Disagree Strongly disagree

**Document (7): Physicians’ Questionnaire**

Section A: Physicians' demographic and clinical practice profile

Instructions: please tick the appropriate answer in the box and fill when necessary

1. **Age**: Years

2. **Gender**:

Male Female

3. **Profession**:

Family Medicine Specialist Medical Officer

**4.** **Current role in the vaccination process:**

Counseling regarding childhood vaccination

Supervision the process of childhood vaccination

Both

Others, please specify: ……………………………………………………...

**5.** **Years of experience** **in childhood vaccination**:

Section B: Physicians' perception regarding the introduction of the fully liquid hexavalent combined vaccine in the national immunization program

**6. I would be interested in introducing the fully liquid hexavalent vaccine instead of the non-fully liquid pentavalent and Hepatitis B vaccines in the national immunization program.**

Strongly agree Agree Neutral

Disagree Strongly disagree

**7. Caregivers/Parents of my pediatric patients would be interested in introducing the fully liquid hexavalent vaccine instead of the non-fully liquid pentavalent and Hepatitis B vaccines in the national immunization schedule.**

Strongly agree Agree Neutral

Disagree Strongly disagree

**8. I believe that the introduction of the fully liquid hexavalent vaccine instead of the non-fully liquid pentavalent and Hepatitis B vaccines in the national immunization schedule could result in cost-savings for the government.**

Strongly agree Agree Neutral

Disagree Strongly disagree

**9. I believe that the introduction of the fully liquid hexavalent vaccine instead of the non-fully liquid pentavalent and Hepatitis B vaccines in the national immunization schedule could reduce the number of patients attending the vaccination facility daily**

Strongly agree Agree Neutral

Disagree Strongly disagree

**10. I believe that introducing the fully liquid hexavalent vaccine instead of the non-fully liquid pentavalent and Hepatitis B vaccines could ease the incorporation of new vaccines such as PCV* into the current immunization schedule.**

Strongly agree Agree Neutral

Disagree Strongly disagree

**11. I believe that introducing the fully liquid hexavalent vaccine instead of the non-fully liquid pentavalent and Hepatitis B vaccines could lead to more compliance from the parent’s side to the immunization schedule.**

Strongly agree Agree Neutral

Disagree Strongly disagree

* PCV: Pneumococcal Conjugated Vaccine

**Document 8: Study Results Tables**

**Table 1: Internal consistency (Reliability) of the study instruments**

| Instrument scale | No. of Items | Cronbach's alpha | Mean | Standard Deviation |
| --- | --- | --- | --- | --- |
| Parents’ fully liquid hexavalent vaccine perception | 5 | 0.910 | 22.01 | 2.421 |
| Nurses’ non-fully liquid vaccine perception | 4 | 0.825 | 15.41 | 3.352 |
| Nurses’ fully liquid hexavalent vaccine perception | 3 | 0.867 | 13.29 | 2.046 |
| Physicians’ fully liquid hexavalent vaccine perception | 6 | 0.918 | 24.98 | 4.511 |

**Table 2: Kaiser-Meyer-Olkin, Bartlett’s Test, communalities, and anti-image correlation for the study scales**

| Instrument scale | Bartlett's test of sphericity (χ2) | Kaiser-Meyer-Olkin (KMO) | df | Sig.* | Communalities (Range) | Anti- Image correlation  (Range) |
| --- | --- | --- | --- | --- | --- | --- |
| Parents’ fully liquid hexavalent vaccine perception | 11156.3 | 0.881 | 10 | <.001 | (0.623-0.826) | (0.838-0.922) |
| Nurses’ non-fully liquid vaccine perception | 182.5 | 0.678 | 6 | <.001 | (0.490-0.830) | (0.634-0.769) |
| Nurses’ fully liquid hexavalent vaccine perception | 155.6 | 0.715 | 3 | <.001 | (0.686-0.848) | (0.660-0.754) |
| Physicians’ fully liquid hexavalent vaccine perception | 227.38 | 0.744 | 15 | <.001 | (0.616-0.842) | (0.689-0.943) |

* χ2= chi-square test.

**Table 3: Summary of total variance explained in exploratory factor analysis (EFA) for study scales**

| Instrument scale | Component | Total | % of Variance | Cumulative % |
| --- | --- | --- | --- | --- |
| Parents’ fully liquid hexavalent vaccine perception | 1 | 3.697 | 73.939 | 73.939 |
| Nurses’ non-fully liquid vaccine perception | 1 | 2.609 | 65.225 | 65.225 |
| Nurses’ fully liquid hexavalent vaccine perception | 1 | 2.377 | 79.222 | 79.222 |
| Physicians’ fully liquid hexavalent vaccine perception | 1 | 4.312 | 71.861 | 71.861 |

Extraction method: Principal Component Analysis

**Table 4: Factor loading based on a principal component analysis for the study scales**

| Item | Perception scale | | | |
| --- | --- | --- | --- | --- |
|  | Parents’ fully liquid hexavalent vaccine | Nurses’ non-fully liquid pentavalent vaccine | Nurses’ fully liquid hexavalent vaccine | Physicians’ fully liquid hexavalent vaccine |
|  | **Component** | | | |
|  | 1 | 1 | 1 | 1 |
| 1 | 0.856 | 0.720 | 0.918 | 0.816 |
| 2 | 0.896 | 0.700 | 0.921 | 0.823 |
| 3 | 0.901 | 0.878 | 0.828 | 0.860 |
| 4 | 0.844 | 0.911 |  | 0.879 |
| 5 | 0.789 |  |  | 0.785 |
| 6 |  |  |  | 0.918 |

**Table 5: Socio-demographic characteristics of parents who participated in the study (n=346)**

| Item | Frequency (%) |
| --- | --- |
| **Parent's Age**  (Mean: 31.8, SD: 5.2)  19-28  29-31  32-35  36-59 | 97 (28)  78 (22.5)  97 (28)  74 (21.4) |
| **Gender**  Female  Male | 244 (70.5)  102 (29.5) |
| **Ethnicity**  Malay  Chinese  Indian  Other | 293 (84.7)  24 (6.9)  24 (6.9)  5 (1.4) |
| **Kinship**  Mother  Father  Family member  Caregiver | 238 (68.8)  101 (29.2)  1 (0.3)  6 (1.7) |
| **Marital status**  Married  Single  Divorced  Widowed | 336 (97.1)  3 (0.9)  3 (0.9)  4 (1.2) |
| **Educational Level**  Primary or less  Secondary  Post-secondary  Missing | 8 (2.3)  146 (42.2)  191 (55.2)  1 (0.3) |
| **Occupation**  Public sector  Private sector  Self-employed  Unemployed  Housewife  Missing | 67 (19.4)  121 (35)  47 (13.6)  14 (4)  90 (26)  7 (2) |

**Table 6: Socio-demographic and professional characteristics of nurses (n=100)**

| Item | Frequency (%) |
| --- | --- |
| **Age (years)**  (Mean: 34.58, SD: 5.59)  25-30  31-34  35-38  39-51 | 30 (30)  22 (22)  24 (24)  24 (24) |
| **Experience (years)**  (Mean:8.39, SD: 5.26)  1-5  6-7  8-10  11-26 | 34 (34)  17 (17)  25 (25)  24 (24) |
| **Gender**  Female  Male | 100 (100)  0 (0) |
| **Subspeciality**  Staff Nurse  Community Health Nurse | 39 (39)  61 (61) |
| **Role**  Prepare and administer vaccine only  Prescribes vaccines only  Prescribe, prepare, and administer the vaccine | 97 (97)  1 (1)  2 (2) |

**Table 7: Socio-demographic and professional characteristics of physicians (n=50)**

| Item | Frequency (%) |
| --- | --- |
| **Age (years)**  (Mean: 32.7, SD: 4.04)  27-30  31-32  33-34  35-47 | 15 (30)  19 (38)  6 (12)  10 (20) |
| **Experience (years)**  (Mean: 4.47, SD: 3.36)  1-3  4-5  6-16  Missing | 25 (50)  13 (26)  11 (22)  1 (2) |
| **Gender**  Female  Male | 44 (88)  6 (12) |
| **Profession**  Medical Officer  Family Medicine Specialist | 100 (100)  0 |
| **Role**  Counseling regarding childhood vaccination  Supervising the process of childhood vaccination  Both counseling and supervision  Missing | 25 (50)  3 (6)  20 (40)  1 (2) |
